# Photoautotrophic microorganisms and bioremediation of industrial effluents: current status and future prospects

**DOI:** 10.1007/s13205-017-0600-5

**Published:** 2017-04-08

**Authors:** Amandeep Brar, Manish Kumar, Vivek Vivekanand, Nidhi Pareek

**Affiliations:** 1 Department of Microbiology, School of Life Sciences, Central University of Rajasthan Bandarsindri, Kishangarh, Ajmer, Rajasthan 305801 India; 2 Centre for Energy and Environment, Malaviya National Institute of Technology, Jaipur, Rajasthan 302017 India

**Keywords:** Biomass, Microorganisms, Photoautotrophs, Remediation, Wastewater

## Abstract

Growth of the industrial sector, a result of population explosion has become the root cause of environmental deterioration and has raised the concerns for efficient wastewater management and reuse. Photoautotrophic cultivation of microorganisms is a boon and considered as a potential biological treatment for remediation of wastewater as it sequesters CO_2_ during growth. Photoautotrophs viz. cyanobacteria, micro-algae and macro-algae can photosynthetically assimilate the excessive pollutants present in the wastewater. The present review emphasizes on the achievability of microorganisms to bestow wastewater as the nutrient source for biomass production, which can further be reused for feed, food and fertilizers. To support this, various case studies have been cited that prove phycoremediation as a cost-effective and sustainable process over conventional wastewater treatment processes that requires high chemical load and more energy inputs.

## Introduction

With the advent of science and technology, the past century had observed the quick growth of various industries, which drastically increased the release of toxic effluents into water bodies. Growth of the industrial sector has become the main cause of environmental pollution and deterioration that includes contamination of soil, sediments, water and air with hazardous and toxic chemicals. Industrial effluents contain a wide range of persistent organic pollutants which are creating disturbance in the ecosystem causing climate changes, reducing ground water levels, melting of icecaps and depletion of the ozone layer due to photochemical oxidation (Varsha et al. 2011). These environmental commotions led to global warming and have compelled the researchers to focus on impacts of pollution and finding measures to reduce it. India has 18% of world’s population while its fresh water resources are only 4% of that available on planet earth. This statistics explains the urge to recycle and use the wastewater due to expected acute water shortage (Malla et al. 2015). Also, the population explosion in urban sector leads to the generation of enormous amount of wastewater and its reuse has been observed as a viable option to cope with the increasing water stress.

The discharge of industrial effluents, i.e. industrial emissions is often well regulated however, accidental release may also be there (e.g. chemicals or oil spills) and contemplated to be persistent and harmful to the aquatic and terrestrial ecosystems. Biogenic or anthropogenic derived compounds are known to play a major role in causing environmental pollution. The prime source of these pollutants is wastewater and solid residues, which are released from a range of industrial activities viz. chemical and pharmaceutical, plastics, paper and pulp mills, textile mills and agriculture etc. The solid residues include phenol, hydrocarbons, dyes, paint effluents, etc. (de Bashan and Bashan 2010). Besides this, the growing urbanization also poses a serious threat to the environment due to release of an extensive amount of domestic and municipal wastewater. Although, it is obligatory to remove most of the nutrients from the wastewater before discharging it into water bodies and in the open land but still not performed in many cases, especially in developing nations (Ruiz-Marin et al. 2010).

Among the ominous impacts of environmental pollution, eutrophication is considered as the most prevalent phenomenon. It is the accumulation of high levels of organic matter and decomposing organisms that deplete the oxygen in the water and cause the death of other organisms. The main cause of eutrophication in natural water has been identified as the nutrients (NH_4_^+^, NO_3_^−^, PO_4_^3−^) present in secondary effluents. These nutrients spoil the quality of water and adversely affects the whole aquatic ecosystem (Gera et al. 2015). Therefore, it is a prerequisite to give a suitable treatment to the wastewater prior to its discharge into waterbodies. Various types of unit processes, i.e. preliminary (which includes primary, secondary and tertiary) treatment, activated sludge (for nitrogen and phosphorous removal), electro-flocculation, membrane-filtration, electro-kinetic coagulation, electro-chemical destruction, ion-exchange, katox treatment (catalytic oxidation) and disinfection (by ozonation, chlorination, ultraviolet light) have been practiced for the removal of nutrients from wastewater. These methods leads to the formation of sludge and are not followed as per the governing standards, thus ultimately cause severe pollution. Moreover, most of the conventional approaches are complicated, expensive and unaffordable in terms of land requirement and energy consumption. The key reasons that slows down the efforts to control pollution in under-developed and developing nations are governed by economic stipulation (Khandare et al. 2013). Apart from this, the conventional wastewater treatment processes that oxidize organic matter present in the effluent are not efficient in removing nitrogen and phosphorus, obstinate organic compounds or heavy metals (Olguin and Sanchez-Galvan 2012). Furthermore, the conventional techniques are environmentally unsustainable as they result in CO_2_ emission and require high chemical load. Improvement in the removal efficiency of classical methods would require an increase of approximately 60–80% in energy consumption and associated costs. Likewise, 20–30% additional electricity costs to an activated sludge is added alone by nitrification (Asano et al. 2007).

## Bioremediation: approaches

Bioremediation is a technology that explores the metabolic potential of microorganisms to clean up the contaminated sites i.e. wastewater, ground or surface waters, soils, sediments, and air in the environment (Boopathy 2000). The process of bioremediation includes detoxification and mineralization that convert waste into inorganic compounds such as carbon dioxide, water and methane. The term bioremediation encompasses both microbial remediation and phytoremediation. Microbial remediation in turn, includes the employment of bacteria, fungi and algae for remediation purposes, thereby involving multiple steps of various enzymatic reactions. In recent years, phycoremediation is evolved as one of the best sustainable way to remove the harmful compounds present in the environment. The main advantage of the photoautotrophic cultivation of algae is the CO_2_ sequestration during cell growth. The nutrients present in the wastewater, rather of being waste, become feed for the algae as utilized and accumulated effectively.

## Phycoremediation

**Fig. 1:**
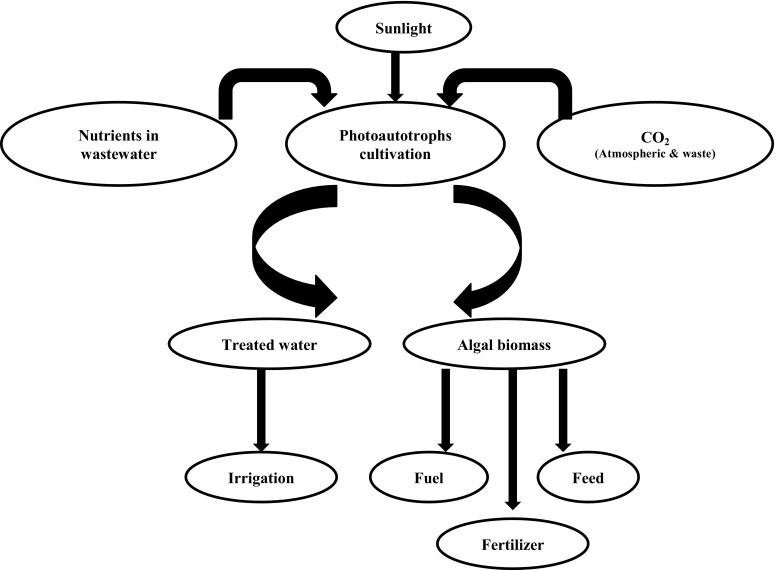
Integrated approach for phycoremediation, energy and fertilizer generation

Phycoremediation is the use of macroalgae, microalgae and cyanobacteria for the removal of nutrients and xenobiotics from wastewater and carbon dioxide from the air (Olguin and Sanchez-Galvan 2012). The photoautotrophic microorganisms are desirable since their use is an eco-friendly process with no secondary pollution as long as the biomass produced is reutilized (Mulbry et al. 2008). Also, the photoautotrophic microorganisms are among one of the most important bio-resources that are currently receiving tremendous popularity due to their ability to grow at a faster rate, the possibility of cultivation on non-arable lands, less water uptake and land requirements (Singh and Olsen 2011). Photoautotrophic metabolism is a process that uses light as a source of energy and converts it into chemical energy through photosynthetic reactions. In this, a large amount of CO_2_ is consumed and converted into biomass, thereby releasing O_2_ into the atmosphere (Pacheco et al. 2015). The biomass generated can further be used to produce biofuels through various pathways i.e. biogas by anaerobic digestion, bio-ethanol by fermentation of carbohydrate and bio-crude oil by high temperature conversion (Fig. 1) (Park et al. 2011).

The repertoire of species being utilized for phycoremediation includes *Chlorella*, *Scenedesmus*, *Phormidium*, *Botryococcus*, *Chlamydomonas*, *Spirulina*, *Oscillatoria*, *Desmodesmus*, *Arthrospira*, *Nodularia*, *Nostoc*, *Cyanothece* etc. (Dubey et al. 2013; Rawat et al. 2011). *Ulva lactuca*, *Kappaphycus alvarezii* etc. are examples of some macroalgae that have been explored for remediation. Additionally, the micro-algae based products can also be used in the production of cosmetics, food or feed additives and fertilizers. A few species of marine algae growing in oceans (e.g. *Haematococcus pluvialis*, *Emiliania huxleyi*, *Dunaliella tertiolecta*) have been optimized for overproduction of products like astaxanthin, β-carotene, omega-3-fatty acids, vitamin-E and pigments (Pangestuti and Kim 2011). Cyanobacteria have shown excellent ability for bioaccumulation and biosorption because they are present ubiquitously in water and soil ecosystems and have a flexible metabolism (Zinicovscaia and Cepoi 2016).

## Carbon assimilation pathways in algae

Algae involves usually three different nutritional modes of carbon assimilation for the synthesis of biomass viz. autotrophic, mixotrophic and heterotrophic (Table 1). For all the metabolic activities of a algal cell, the photosynthetically fixed carbon dioxide, in the form of glucose serves as energy source (Chang et al. 2011).

**Table 1:** Carbon assimilation modes, growth patterns and affecting factors in algae

Factors	Autotrophic	Heterotrophic	Mixotrophic
Light	Required	Required/not	Required/not
Source of carbon	CO_2_ fixation	CO_2_ fixation/organic carbon	CO_2_ fixation/extra-cellular organic carbon
Pattern of growth	Increase in daylight; decrease at night; as autotrophic input of metabolites ceases at night	Remains constant during both day and night as carbon and nutrients are available continuously	Remains constant as independent from both photosynthesis and growth substrates
Source of ATP production	Only light	Either light or glucose	Both light and glucose
Algal system operated	Open/raceway ponds	Photo-bioreactors	Fed-batch

Large scale microalgal cultivation systems (i.e. open/raceway ponds) are usually operated under photo-autotrophic conditions (Mata et al. 2010). However, these photo-autotrophic systems require long period of cultivation and resulted in low biomass concentration i.e. *Chlorella fusca* var. *vacuolata*, *Scenedesmus obliqus* and *Anabaena variabilis* (Pacheco et al. 2015). Some microalgae viz. *Chlorella*, *Scenedesmus*, *Tetraselmis* and *Nitzschia* have potential to switch between autotrophic and heterotrophic mode of assimilation with variable environmental conditions. The heterotrophic cultivation may be hampered due to the deficiency of organic carbon in the surrounding as organic compounds acts as a sole source of carbon and energy in dark condition and microalgae prefers autotrophic mode of nutrition. The heterotrophic nutrition can occur both in the presence (photo-heterotrophic condition) or absence of light and the growth of alga remains static as observed in different species of *Chlorella* and *Nostoc*. For large scale photo-bioreactors, the main hindrance of light dependency is escaped which is required to achieve a high cell density in photo-heterotrophic mode of nutrition (Mohan et al. 2015). According to Perez-Garcia et al. (2011) heterotrophic mode of nutrition is cost-effective, easy and simple to support the cultivation of microalgae at large scale as compared to autotrophic cultivation. *Nannochloropsis* sp., *Rhodomonas reticulate*, *Cyclotella cryptica* exhibit mixotrophic mode of nutrition is a deviation of heterotrophic system where organic compounds and carbon-dioxide are assimilated together. This mode shows reduced photo-inhibition and has improved growth rates as compared to the autotrophic and heterotrophic means.

## Factors affecting algal performance in remediation

The wastewater usually contains organic carbon, nitrogen, phosphorous and other compounds, making it suitable for the cultivation of algae. The efficiency of algae to grow in wastewater depends upon various critical variables including pH, temperature, availability of light, CO_2_, O_2_ and especially nutrient concentrations. Primary settled sewage water notably supported microalgal growth under long photoperiods with addition of CO_2_ but with increase in temperature, the algal biomass decreased (Pittman et al. 2011). It has been shown that the preconditioned starvation of microalgae would lead to increased uptake of nitrogen and hence, wastewater will be remediated within very short period of time (Rawat et al. 2011). Nitrogen present in the form of ammonia in higher concentrations may inhibit the algal growth. The presence of heavy metals, toxic organic compounds and other biotic factors such as bacterial pathogens and zooplanktons also inhibit the growth of algae and this issue is peculiar with industrial wastewater. Suspended solids affects the turbidity, which in turn affects the algal growth, as light is an important factor.

## Advantages of phycoremediation

Phycoremediation is a cost-effective, eco-friendly and comparatively safe process. It can effectively reduce the nutrient load of wastewater thereby reducing total dissolved solids. The microorganisms involved are non-pathogenic, do not produce harmful by-products and elevate the dissolved oxygen levels by photosynthesis. The operation and maintenance of phycoremediation setup are simple. Also, it reduces sludge formation, keeps the bacterial population under check and removes CO_2_ from the waste contributing to the reduction of green house gases. The algal biomass produced in this process has high nutrient value and is suitable to use both as a feed and fuel after proper bioprocessing (Rawat et al. 2011; Abdelaziz et al. 2014; Parjo and Razak 2015).

## Existing biological treatment systems vs phycoremediation

Wastewater treatment is an important push for the advancement of mankind. Conventional wastewater treatment methods are broadly classified into physical, chemical and biological methods. In case of physical methods, mechanical forces are applied to remove contaminants and this step forms the base for wastewater treatment processes. Physical methods also include adsorption and common adsorbents used are activated carbon, peat, wood chips and silica gels. These sorbents are expensive and cannot be used for complex wastewater treatment processes. The major disadvantage of using physico-chemical methods viz. coagulation, flocculation, ion-exchange or membrane filtration is the formation of enormous quantities of chemical sludge whose disposal presents another challenge (Zinicovscaia and Cepoi 2016). The chemical processes are destined to treat by chemical reactions (as ozonation, disinfection or dechlorination) and are always used in conjunction with physical unit operations and biological processes. A biological treatment process usually includes anaerobic digestion with the help of indigenous microorganisms. This process involves use of trickling filters or rotating biological contactors. Wastewater treatment processes employing trickling filters are convenient for operation and maintenance. Also, they are considered as energy savior than activated sludge processes (example: Fenton’s reagent which is a chemical process) as they do not require aeration. However, their application decreased with time as they are not efficient in removing high suspended solids and BOD of wastewater. Although, employment of aforementioned technologies have proved wide application, but they have certain limits (Molof and Kim 1997). With increase in the environmental pollution, the need has been raised to search for natural remediation methods (Madigan et al. 1997; Maier et al. 2009). Whereas phycoremediation have the inherent efficacy to treat wastewater by utilizing nutrients and metals from the wastewater. This technique has the prospective for its use as an alternative biomass source for bioenergy production. Expedition of the usefulness of biological wastewater treatment by microalgae coupled to biofuel production, is further made significant as a result of increase in global warming, depletion of fossil fuels and the need for the maintenance of green-house gases (Rawat et al. 2011).

## Application of photoautotrophic microorganisms—case studies

The release of industrial effluents poses a serious threat to the receiving water bodies (de Bashan and Bashan 2010). This global problem can be solved by the use of photoautotrophic microorganisms where the wastewater is used as a feed for microbial growth and in turn, the microorganisms will be removing excess of nutrients present (Pittman et al. 2011). Photoautotrophic microorganisms supply the molecular oxygen to heterotrophic partners thereby accomplishing nutrient removal with net energy savings in the wastewater treatment system. Hence, it supports the initial steps in the process of biodegradation (Perez-Garcia et al. 2011).

### Leather processing unit

*Chlorella vulgaris* was employed to treat the effluent of the leather-processing manufacturing chemical facility (Hanumantha Rao et al. 2011), that contains heavy metals, chemicals, residual pigments and casein. Existing effluent treatment plant (ETP) of the industry, converted the pollutants into solid waste by polyelectrolyte precipitation and pressure filtration that led to accumulation of tons of solid waste over years. Phycoremediation of effluent showed significant reduction in calcium and magnesium levels up to 63 and 50%, respectively. Also, free ammonia, nitrite, BOD and COD levels were reduced by 80, 89, 22 and 38%, respectively. Phycoremediation of ETP solid waste resulted in complete removal of the offensive smell of the sample. Also, the color of waste was changed from black to green and total dissolved solids were removed by 14%.

### Carpet mill effluent

Chinnasamy et al. (2010) studied the remediation of wastewater generated from carpet mills along with the sewage from the Dalton area in North Central Georgia. The samples were subjected to the blooming process by incubating in a growth chamber and native algal strains were isolated along with a consortium of the same. The isolates included green algal species viz. *Chlorella*, *Chlamydomonas*, *Scenedesmus*, *Gloeocystis* and cyanobacterial species, i.e. *Anabaena* and *Limnothrix*. The researchers showed that the consortium could produce ~28 tons of biomass and ~3830 L of oil ha^−1^ year^−1^. Study of nutrient dynamics showed the depletion of nitrate-N up to 99%, ammonia-N to 100% and phosphate-P up to 75%. In 72 h, nitrate-N was removed to 99.7–99.8% and phosphate-P removal reached 98.8–99.9% at ambient air and 96.5% under elevated CO_2_ (6%) levels. The research concluded that consortium of native algal strains removed >96% nutrients in 72 h.

### Dairy manure effluents

The dairy effluent was collected from Madavaram dairy plant, Chennai, India and it largely contains a greater concentration of milk constituents viz. casein, lactose, fat and high amount of BOD and COD. In this study, employment of *Nostoc* sp. decreased the total reduced solids to 53.93%, total dissolved solids to 20.21%, alkalinity to 18.13% and phosphate content to 21.08% in the effluent. Also, BOD and COD levels were reduced to 40.25 and 44.44%, respectively. The study supported utilization of green algae as efficient, cost-effective and eco-friendly approach for treatment of dairy effluent (Kotteswari et al. 2012). Filamentous green algae were grown in outdoor raceways at different loading rates having raw and anaerobically digested dairy manure effluent in order to study the nutrient content and nutrient recovery (Mulbry et al. 2008). The algal biomass was harvested after 4–12 days depending upon the loading rate and algal growth. The research concluded that the mean algal nitrogen and phosphorous increased approximately by two fold and this algal biomass can serve as a feed supplement or can act as a slow-release fertilizer.

### Textile effluent

Microalga *Chlorella pyrenoidosa* was used to study the phycoremediation of textile wastewater (Pathak et al. 2014). The alga was efficiently removing the nutrient load from the effluent as nitrate and phosphate were reduced by 62 and 87%, respectively. The organic load was also reduced by 81% in the effluent. The results suggested that alga has an enormous potential for treatment of textile effluent as an economic and effective adsorbent. *Chlorella vulgaris* was also studied for the remediation of textile wastewater. The study showed that the algal species remove color either by adsorption or by bioconversion ranged from 41.8 to 50.0% in HRAP (high rate algal ponds). They concluded that the growth of *Chlorella* not only reduces the pollutant load (COD, NH_4_-N, PO_4_-P) but also the color during remediation of textile wastewater (Lim et al. 2010).

### Agro-industrial wastewater

Wastewater from potato processing industry and treated liquid fraction of pig manure were treated using two combined processes employing *Chlorella sorokiniana* and aerobic bacteria in 5 L photobioreactors (Hernández et al. 2013). Total COD removal recorded was 62.3% for treated liquid fraction of pig manure and 84.8% for potato processing industry. Ammonium was removed up to 82.7% in treated liquid fraction of pig manure and was almost exhausted in potato processing industry. Photobioreactor with treated liquid fraction of pig manure had more than 75% nitrate removal efficiency. In treated liquid fraction of pig manure, the total solid removal efficiency increased from 12.2 to 21.3% when the substrate/inoculum ratio was increased from 0.5 to 2. For the same changes in potato processing industry, the efficiency was increased from 15.8 to 25%.

### Municipal wastewater

Municipal wastewater was collected from various stages of the treatment process and was analyzed simultaneously for its ability to support the growth of *Chlorella* sp. and treatment. Total phosphorous has been removed by 79.0 and 80.9% in autoclaved and non-autoclaved samples. The concentration of COD decreased drastically from 2390 to 230 mg L^−1^ in the autoclaved sample and from 2304 to 210 mg L^−1^ in the non-autoclaved sample within first two days of cultivation and by the completion of the experiment, 90.3 and 90.8% of COD was removed. More than 93% of NH_4_-N and 89% of total nitrogen was removed by algal treatment (Li et al. 2011).

### Heavy metal

Chromium, a toxic metal is present in the effluents of dye, tanning, paper-pulp, printing and the electroplating industries and is carcinogenic and mutagenic. Yewalkar et al. (2007) concluded that the disappearance of Cr(VI) from the medium was the result of reduction by live algal cells of *Chlorella* sp., since the cell supernatant did not show this activity. This reduction was not merely due to absorption of chromium in the cells as studies showed the cells retained only 10–21% of total Cr(VI). Colorimetric assay showed >50% reduction in the Cr(VI) concentration under similar conditions which may be due to the conversion of Cr(VI) to Cr(III) by *Chlorella* sp. *Nitella* sp. can also be effectively used to remediate Cr(VI) contaminated water either passively or actively in wetland systems, depending upon the concentration of Cr(VI) (Gomes and Asaeda 2009). Various cyanobacterial species (*Nostoc muscorum*, *Spirulina platensis* and *Aphanothece flocculosa*) have shown the biosorption ability towards metal ions (Cu^2+^, Cd^2+^, Cr^3+^, Cr^6+^, Co^2 +^, Ni^2+^ and Fe^3+^) (Zinicovscaia and Cepoi 2016).

## Phycoremediation: challenges

There are ample evidences available that support the phycoremediation as an efficient technique to remediate the different types of wastewater. However, this has to be confirmed under the absolute local environmental conditions since there are many factors (like optimum pH, salinity, composition of wastewater, etc.) which are variable but still define the growth of algae. There is a need for the active integration of practical approaches with respect to the availability of wastewater treatment with industrial effluents. There are many challenges in the large scale production of algae in wastewater that need to be addressed yet. Also, it is necessary to prove the ultimate economic viability of the process. Most of the algae based reactor development studies are confined to laboratory scale, and their industrial or large scale execution is still in its infancy. Up-scaling of the process further presents challenges before environmental microbiologists with respect to organism employed, growth requirements and efficiency. For example, in laboratory scale studies, glucose is usually added as a carbon source in biological treatment systems while, additional carbon supplementation becomes a limiting factor at large scale. So, there is a need to design a cost-effective bioreactor as well as to choose species that can grow efficiently in a low-cost open system without having any impact of the contaminants and the competing species. The research on phycoremediation needs to conquer a number of constraints before it can be extended for a large commercial setup. The key aspects viz. productivity of algal culture, nutrient uptake and growth, space, dissolved oxygen, biomass harvesting etc. requires urgent diligence. These challenges are terrifying, but there are many reasons to be optimistic.

## Conclusion and future prospects

Employment of green algae for remediation presents an effective approach to alter the physicochemical parameters of wastewater and reducing the nutrient load. For wastewater treatment and bioremediation, integrated algal systems may be used to capture carbon, nitrogen and phosphorus from industrial, municipal and agricultural wastes thus minimizing the negative effects of CO_2_ and water pollution. The high biomass productivity of microalgae grown in wastewater suggests that this method provides a real potential for the production of a sustainable environment. Usually, wastewater treatment involves additional cost, but if the treatment itself becomes a value-adding process by preventing the pollution and helps to meet the environmental standards, it enhances the profitability and increases the sustainability of the industry. This review laid emphasis on the exploitation of photoautotrophic microorganisms for the treatment of industrial effluents as they have simple cell structures and is able to survive and may reproduce in almost all the environmental conditions and contaminants with the effective reduction of pollutant load.

The major challenges in the near future are for balancing the needs of algal growth along with increased production costs and higher-value utilization of the produced algal biomass. The various problems associated with large-scale algal systems include lack of control, contamination, loss of water by evaporation, and irregular productivities. Thus, research should focus to minimize these drawbacks and in this line operating cost-effective photo-bioreactor is also a challenge.

However, phycoremediation is a proficient method for which biomass recovery is essential as it is during this step; the large operational and implementation costs are found. Integrated technology may be developed which can solve the problem of remediation of wastewater and energy crisis. Therefore, the effectiveness of the application of photoautotrophic microorganisms depends on the development of an integrated process for algal biomass production in the wastewater and use of biomass for the production of commercially valuable products viz. fuel, feed and fertilizers.

## References

[CR1] Abdelaziz AE, Leite GB, Belhaj MA, Hallenbeck PC (2014). Screening microalgae native to Quebec for wastewater treatment and biodiesel production. Bioresour Technol.

[CR2] Asano T, Burton F, Leverenz H, Tsuchihashi R, Tchobanoglous G (2007). Water reuse.

[CR3] Boopathy R (2000). Factors limiting bioremediation technologies. Bioresour Technol.

[CR4] Chang RL, Ghamsari L, Manichaikul A, Hom EF, Balaji S, Fu W, Shen Y, Hao T, Palsson BØ, Salehi-Ashtiani K (2011). Metabolic network reconstruction of *Chlamydomonas* offers insight into light-driven algal metabolism. Mol Syst Biol.

[CR5] Chinnasamy S, Bhatnagar A, Hunt RW, Das KC (2010). Microalgae cultivation in a wastewater dominated by carpet mill effluents for biofuel applications. Bioresour Technol.

[CR6] de Bashan LE, Bashan Y (2010). Immobilized microalgae for removing pollutants: review of practical aspects. Bioresour Technol.

[CR7] Dubey SK, Dubey J, Mehra S, Tiwari P, Bishwas A (2013). Potential use of cyanobacterial species in bioremediation of industrial effluents. Afr J Biotechnol.

[CR8] Gera G, Yewalkar S, Nene S (2015). Algal biorefinery: an integrated approach.

[CR9] Gomes PI, Asaeda T (2009). Phycoremediation of Chromium (VI) by *Nitella* and impact of calcium encrustation. J Hazard Mater.

[CR10] Hanumantha Rao P, Ranjith Kumar R, Raghavan B, Subramanian V, Sivasubramanian V (2011). Application of phycoremediation technology in the treatment of wastewater from a leather-processing chemical manufacturing facility. Water Sa.

[CR11] Hernández D, Riaño B, Coca M, García-González MC (2013). Treatment of agro-industrial wastewater using microalgae–bacteria consortium combined with anaerobic digestion of the produced biomass. Bioresour Technol.

[CR12] Khandare RV, Kabra AN, Kadam AA, Govindwar SP (2013). Treatment of dye containing wastewaters by a developed lab scale phytoreactor and enhancement of its efficacy by bacterial augmentation. Int Biodeter Biodegr.

[CR13] Kotteswari M, Murugesan S, Ranjith Kumar R (2012). Phycoremediation of dairy effluent by using the microalgae *Nostoc* sp.. Int J Environ Res Dev.

[CR14] Li Y, Chen YF, Chen P, Min M, Zhou W, Martinez B, Zhu J, Ruan R (2011). Characterization of a microalga *Chlorella* sp. well adapted to highly concentrated municipal wastewater for nutrient removal and biodiesel production. Bioresour Technol.

[CR15] Lim SL, Chu WL, Phang SM (2010). Use of *Chlorella vulgaris* for bioremediation of textile wastewater. Bioresour Technol.

[CR16] Madigan MT, Martinko JM, Parker J (1997). Biology of microorganism.

[CR17] Maier RM, Pepper IL, Gerba CP (2009). Environmental microbiology.

[CR18] Malla FA, Khan SA, Sharma GK, Gupta N, Abraham G (2015). Phycoremediation potential of *Chlorella minutissima* on primary and tertiary treated wastewater for nutrient removal and biodiesel production. Ecol Eng.

[CR19] Mata TM, Martins AA, Caetano NS (2010). Microalgae for biodiesel production and other applications: a review. Renew Sust Energ Rev.

[CR20] Mohan SV, Rohit M, Chiranjeevi P, Chandra R, Navaneeth B (2015). Heterotrophic microalgae cultivation to synergize biodiesel production with waste remediation: progress and perspectives. Bioresour Technol.

[CR21] Molof AH, Kim S (1997). US Patent No. 5,651,891.

[CR22] Mulbry W, Kondrad S, Pizarro C, Kebede-Westhead E (2008). Treatment of dairy manure effluent using freshwater algae: algal productivity and recovery of manure nutrients using pilot-scale algal turf scrubbers. Bioresour Technol.

[CR23] Olguin EJ, Sanchez-Galvan G (2012). Heavy metal removal in phytofiltration and phycoremediation: the need to differentiate between bioadsorption and bioaccumulation. N Biotechnol.

[CR24] Pacheco MM, Hoeltz M, Moraes MS, Schneider RC (2015). Microalgae: cultivation techniques and wastewater phycoremediation. J Environ Sci Health Tox Hazard Subst Environ Eng.

[CR25] Pangestuti R, Kim SK (2011). Biological activities and health benefit effects of natural pigments derived from marine algae. J Funct Foods.

[CR26] Parjo K, Razak RA (2015). Phycoremediation of wastewaters and potential hydrocarbon from microalgae: a review. Adv Environ Biol.

[CR27] Park J, Craggs R, Shilton A (2011). Wastewater treatment high rate algal ponds for biofuel production. Bioresour Technol.

[CR28] Pathak VV, Singh DP, Kothari R, Chopra AK (2014). Phycoremediation of textile wastewater by unicellular microalga *Chlorella pyrenoidosa*. Cell Mol Biol.

[CR29] Perez-Garcia O, Escalante FM, de Bashan LE, Bashan Y (2011). Heterotrophic cultures of microalgae: metabolism and potential products. Water Res.

[CR30] Pittman JK, Dean AP, Osundeko O (2011). The potential of sustainable algal biofuel production using wastewater resources. Bioresour Technol.

[CR31] Rawat I, Kumar RR, Mutanda T, Bux F (2011). Dual role of microalgae: phycoremediation of domestic wastewater and biomass production for sustainable biofuels production. Appl Energy.

[CR32] Ruiz-Marin A, Mendoza-Espinosa LG, Stephenson T (2010). Growth and nutrient removal in free and immobilized green algae in batch and semi-continuous cultures treating real wastewater. Bioresour Technol.

[CR33] Singh A, Olsen SI (2011). A critical review of biochemical conversion, sustainability and life cycle assessment of algal biofuels. Appl Energy.

[CR34] Varsha Y, Naga Deepthi CH, Chenna S (2011). An emphasis on xenobiotic degradation in environmental clean up. J Bioremed Biodegrad.

[CR35] Yewalkar SN, Dhumal KN, Sainis JK (2007). Chromium (VI)-reducing *Chlorella* spp. isolated from disposal sites of paper-pulp and electroplating industry. J Appl Phycol.

[CR36] Zinicovscaia I, Cepoi L (2016). Cyanobacteria for bioremediation of wastewaters.

